# The Predictor of Mortality within Six-Months in Patients with Spontaneous Cerebellar Hemorrhage: A Retrospective Study

**DOI:** 10.1371/journal.pone.0132975

**Published:** 2015-07-17

**Authors:** Chih-Ya Chang, Ching-Yueh Lin, Liang-Cheng Chen, Chia-Hung Sun, Tsung-Ying Li, Tung-Han Tsai, Shin-Tsu Chang, Yung-Tsan Wu

**Affiliations:** 1 Department of Physical Medicine and Rehabilitation, Tri-Service General Hospital, No. 325, Sec. 2, Neihu District, Taipei, Taiwan; 2 Department of Physical Medicine and Rehabilitation, School of Medicine, National Defense Medical Center, Taipei, Taiwan; 3 Department of Neurosurgery, Tri-Service General Hospital, National Defense Medical Center, Taipei, Taiwan; 4 Department of Rehabilitation, Taichung Veterans General Hospital, Taichung, Taiwan; St Michael's Hospital, University of Toronto, CANADA

## Abstract

**Background and Purpose:**

The mortality rate of cerebellar hemorrhage (CH) is generally higher than other types of intracranial hemorrhage. Recently, the increased survey rate of CH has come from improved clinical imaging and earlier surgical intervention. Hence, the predictors of intermittent- (1 to 6 months) and long-term (> 6months) mortality are clinically practical use for educational and therapeutic decisions. Unfortunately, the factors predictive mortality within six-month had not yet been systematically investigated.

**Methods:**

Seventy-two patients with acute spontaneous CH were retrospectively analyzed. The patients were divided into the six-month mortality group (n = 21, died within 6 months after CH onset) and survival group (n = 51, survived beyond 6 months). The independent predictors of six-month mortality were investigated by multivariate Cox proportional hazards regression.

**Results:**

The radiological brainstem compression (hazard ratios = 23.5; p < 0.001) was independent predictor of mortality within six-month after CH onset. The median onset time of six-month mortality was 5 days in patients with brainstem compression (p < 0.001) and the hazard ratios for the onset time was 13.1 compared with those without brainstem compression (95% CI, 4.7 to 36.3, p < 0.001).

**Conclusions:**

We report the first study that radiological brainstem compression predicted the mortality within six-month after onset of CH. Patients with radiological brainstem compression were about 23 times more likely to die within 6 months after CH than those without radiological brainstem compression.

## Introduction

Cerebellar hemorrhage (CH) is the least common type of intracranial hemorrhage (ICH), accounting for 10% of all ICH cases in western countries [1]. Moreover, the mortality rates for patients with CH, ranging from 20% to 75%, were generally higher than other types of ICH in different regions, perhaps due to heterogeneous management strategies and observation periods [1, 2]. In recent years, the largest contributions to the decreased mortality rate had come from improved clinical imaging and early surgical intervention to relieve brainstem compression and hydrocephalus, which were major risk factors for a rapidly worsening prognosis in the acute stage. Hence, the predictors for early (1 month), intermediate (1 to 6 months) and long term mortality (> 6 months) in patients with spontaneous CH are clinically valuable. In previous studies, we surveyed that blood glucose/sugar (BS) levels 140 mg/dl upon arrival at the hospital and maximum hematoma diameter 3 cm were strong predictive factors of poor outcomes at discharge in patients with acute spontaneous CH [3]. Furthermore, Glasgow Coma Scale (GCS) scores 8 upon hospital arrival and radiological brainstem compression were strong predictors of first-week mortality [4]. Among patients of CH in the sub-acute and chronic stages, the associated predictors of intermediate- or long-term mortality may be of practical use for educational and therapeutic decisions. However, most studies associated predictive factors of clinical outcome in the sub-acute or chronic stages survey the follow-up period within 6 months after onset of CH [5]. Only 3 studies included data beyond 6 months [6–8]. Unfortunately, the predictive factors of mortality within six-month have not yet been systematically investigated. The purpose of this study was to survey the significant predictors of mortality within six-month in patients with spontaneous CH.

## Methods

This retrospective study was approved by the Institutional Review Board (IRB)/Ethics Committee of Tri-Service General Hospital, National Defense Medical Center, Taiwan (No. 100-05-104). Between August 2004 to May 2012, 72 consecutive patients, diagnosed of acute spontaneous CH without prior disability and admitted to our hospital within 48 hours following onset, were collected, reviewed, de-identified by the authors, and analyzed anonymously. The authors had access to the patients’ records prior to data anonymization, and the IRB/Ethics Committee waived the need for informed consent. None of the patients had a prior stroke or complications associated with another hemorrhagic lesion or hemorrhagic transformation of ischemic stroke.

On admission, all patients underwent detailed physical examinations, routine laboratory tests, and imaging. The initial neurological state was evaluated by the GCS. The patients, blood sugar (BS), systolic blood pressure (SBP), diastolic blood pressure (DBP), heart rate (HR), and body temperature (BT) were measured immediately before they underwent computer tomography. Basic characteristics, including age, gender, date of death, and pre-existing or concomitant illnesses, were collected. Coagulopathy were defined by an international normalized ratio > 1.4, partial thromboplastin time > 35, and platelet count > 100,000 [9]. Image findings, including the maximum diameter of hematoma, evidence of intraventricular hemorrhage (IVH), radiographic signs of brainstem compression (presence of obliterated basal cisterns), and hydrocephalus, were also obtained.

In our center, patients with hematoma < 3cm in diameter, and no hydrocephalus or brainstem compression were candidates for conservative treatments. Otherwise, surgical decompression consist of craniotomy and evacuation of hematoma were performed immediately in patients with neurologic deterioration or brainstem compression and/or hydrocephalus from ventricular obstruction (except patients who had a large hemorrhage or coma status without brainstem reflexes due to its predictably poor outcome) [10]. All the above information was acquired by retrospective analysis of patient charts and clinical data concerning death during the follow-up period were retrieved from medical records. All the patients were followed for at least 6 months.

Statistical analyses were performed with SPSS 13.0 for Windows. To determine the independent predictors of mortality within six-month, univariate and multivariate Cox proportional hazards regression models were used. The cut-off points of continuous variables for predicting mortality within six-month were based on previously published cut-off values: age 65 [11], BS levels 140 mg/dl [3, 10], maximum diameter 3 cm [3, 12], SBP 200 mmHg [3, 12], GCS scores 8 [3, 5], HR 100 beats/min [12], BT 37.5C [3, 13], and DBP 120 mmHg [3, 14]. Variables showing p < 0.05 in the univariate analysis were incorporated into the multivariate analyses, which were performed using the forward regression method. The independent risk factors in the final model were presented as hazard ratios (HRs) with 95% confidence intervals (CIs). The rate of six-month mortality between brainstem and no brainstem compression groups was compared by using Kaplan-Meier method and the log-rank test. Moreover, Cox regression was used to estimate HRs and 95% CIs for six-month mortality between these two groups. Statistical significance was set at p < 0.05.

## Results

The baseline characteristics of the study population and the potential factors affecting six-month mortality were summarized in Tables 1 and 2. In total, 21 (29.1%) patients died within 6 months (11 men and 10 women) and 51 (70.9%) patients survived (33 men and 18 women). The mean age of the patients was 65.2 ± 16.1 years (standard deviation). Forty-four (61.6%) of the 72 patients were men. The initial mean GCS score was 10.8 ± 4.7 and BS levels, 168.7 ± 57.4 mg/dl. The mean maximum diameter of the hematoma was 3.7 ± 1.6 cm. Total 31 patients (43%) received surgical intervention, 24 patients (47%) in survival group and 7 patients (33.3%) in six-month mortality group. Other radiological findings included hydrocephalus in 50 patients (69.4%), and radiological brainstem compression in 22 patients (30.6%).

**Table 1:** The baseline characteristics of the study population affecting six-month mortality.

Variable	All patients (n = 72)	Survival group (n = 51)	Six-month mortality (n = 21)
Age (year) (SD)	65.2 ± 16.1	64.5 ± 15.6	67 ± 17.5
Initial GCS (SD)	10.8 ± 4.7	12.1 ± 4	7.5 ± 4.8
Initial SBP (mmHg) (SD)	190.3 ± 39	185.9 ± 37.1	201.2 ± 42.4
Initial DBP (mmHg) (SD)	104.5 ± 25	105 ± 24	103.4 ± 27.9
Initial HR (beat/min) (SD)	89.2 ± 19	87.5 ± 20.6	93.3 ± 14
Initial BT (°C) (SD)	36.4 ± 0.8	36.5 ± 0.8	36.1 ± 0.9
Maximum diameter (cm) (SD)	3.7 ± 1.6	3.4 ± 1.5	4.6 ± 1.6
Initial BS (SD)	168.7 ± 57.4	154.8 ± 48.5	202.2 ± 64.5

Note: GCS = Glasgow Coma Scale, SBP = systolic blood pressure, DBP = diastolic blood pressure, HR = heart rate, BT = body temperature, BS = blood glucose/sugar, SD = standard deviation.

**Table 2:** The multivariate Cox regression analysis of the study population and the potential factors affecting six-month mortality.

Variable	All patients (n = 72)	Survival group (n = 51)	Six-months mortality (n = 21)	Univariate analysis		Multivariate analysis	
				Hazard ratio (95% CI.)	*p* value	Hazard ratio (95% CI.)	*p* value
Male sex, n (%)	44 (61.1)	33 (64.7)	11(52.4)	0.6 (0.2–1.7)	0.330		
Age (year) ≥65	42 (58.3)	29 (56.9)	13 (61.9)	1.2 (0.4–3.5)	0.693		
Smoking, *n* (%)	31 (43.1)	21 (41.2)	10 (47.6)	1.3 (0.5–3.6)	0.616		
Drinking, *n* (%)	24 (33.3)	15 (29.4)	9 (42.9)	1.8 (0.6–5)	0.296		
Past medical history							
Hypertension, *n* (%)	51 (70.8)	36 (70.6)	15 (71.4)	1 (0.3–3.2)	0.943		
Diabetes mellitus, *n* (%)	20 (27.8)	11 (21.6)	9 (42.9)	2.7 (0.9–8.1)	0.067		
Coronary artery disease, *n* (%)	21 (29.2)	12 (23.5)	9 (42.9)	2.4 (0.8–7.2)	0.101		
Dyslipidemia, *n* (%)	2 (2.8)	2 (3.9)	0 (0)	-	0.357		
Coagulopathy, *n* (%)	8 (11.1)	3 (5.9)	5 (23.8)	5 (1.1–23.3)	0.028*		
Clinical parameters							
Initial GCS ≤8	25 (34.7)	11 (21.6)	14 (66.7)	7.3 (2.4–22.4)	0.000***		
Initial SBP (mmHg) ≥200	27 (37.5)	18 (35.3)	9 (42.9)	1.4 (0.5–3.9)	0.547		
Initial DBP (mmHg) ≥120	18 (25)	13 (25.5)	5 (23.8)	0.9 (0.3–3)	0.881		
Initial HR (beat/min) ≥100	22 (30.6)	15 (29.4)	7 (33.3)	1.2 (0.4–3.6)	0.743		
Initial BT (°C) ≥37.5°C	5 (6.9)	5 (9.8)	0 (0)	-	0.137		
Initial BS ≥140	44 (61.1)	25 (49)	19 (90.5)	9.9 (2.1–46.9)	0.001**		
Radiological parameters							
Maximum diameter (cm) ≥3	41 (56.9)	23 (45.1)	18 (85.7)	7.3 (1.9–27.9)	0.002**		
Hydrocephalus, n (%)	50 (69.4)	32 (62.7)	18 (85.7)	3.6 (0.9–13.7)	0.054		
Brainstem compression, *n* (%)	22 (30.6)	6 (11.8)	16 (76.2)	24 (6.4–89.6)	0.000***	23.5 (6.3–87.6)	0.000***
IVH, *n* (%)	44 (61.1)	28 (54.9)	16 (76.2)	2.6 (0.8–8.3)	0.092		
Surgical intervention	31 (43)	24 (47)	7 (33.3)	6.0 (0.2–1.5)	0.268		

Note: GCS = Glasgow Coma Scale, SBP = systolic blood pressure, DBP = diastolic blood pressure, HR = heart rate, BT = body temperature, BS = blood glucose/sugar, IVH = intraventricular hemorrhage.

Variables associated with p value<0.05 on univariate analysis were incorporated in the multivariate analysis which was performed by forward Cox regression model.

The independent risk factors in the final model were presented as hazard ratios, including 95% confidence intervals (CI).

*** p ≦ 0.001

** p ≦ 0.01

* p ≦ 0.05.

The results from the univariate and multivariate forward Cox regression analyses for six-month mortality were shown in Table 2. The following 5 risk factors were significantly correlated with six-month mortality in the univariate analysis: coagulopathy (HRs = 5; p = 0.028), GCS scores 8 (HRs = 7.3; p < 0.001), initial BS levels 140 mg/dl (HRs = 9.9; p = 0.001), maximum hematoma diameter 3 cm (HRs = 7.3; p = 0.002), and radiological brainstem compression (HRs = 24; p < 0.001). However, only radiological brainstem compression (HRs = 23.5; 95% CI, 6.3–87.6; p < 0.001) was shown to be an independent predictor of mortality within six-month in the multivariate Cox logistic regression.

**Fig 1:**
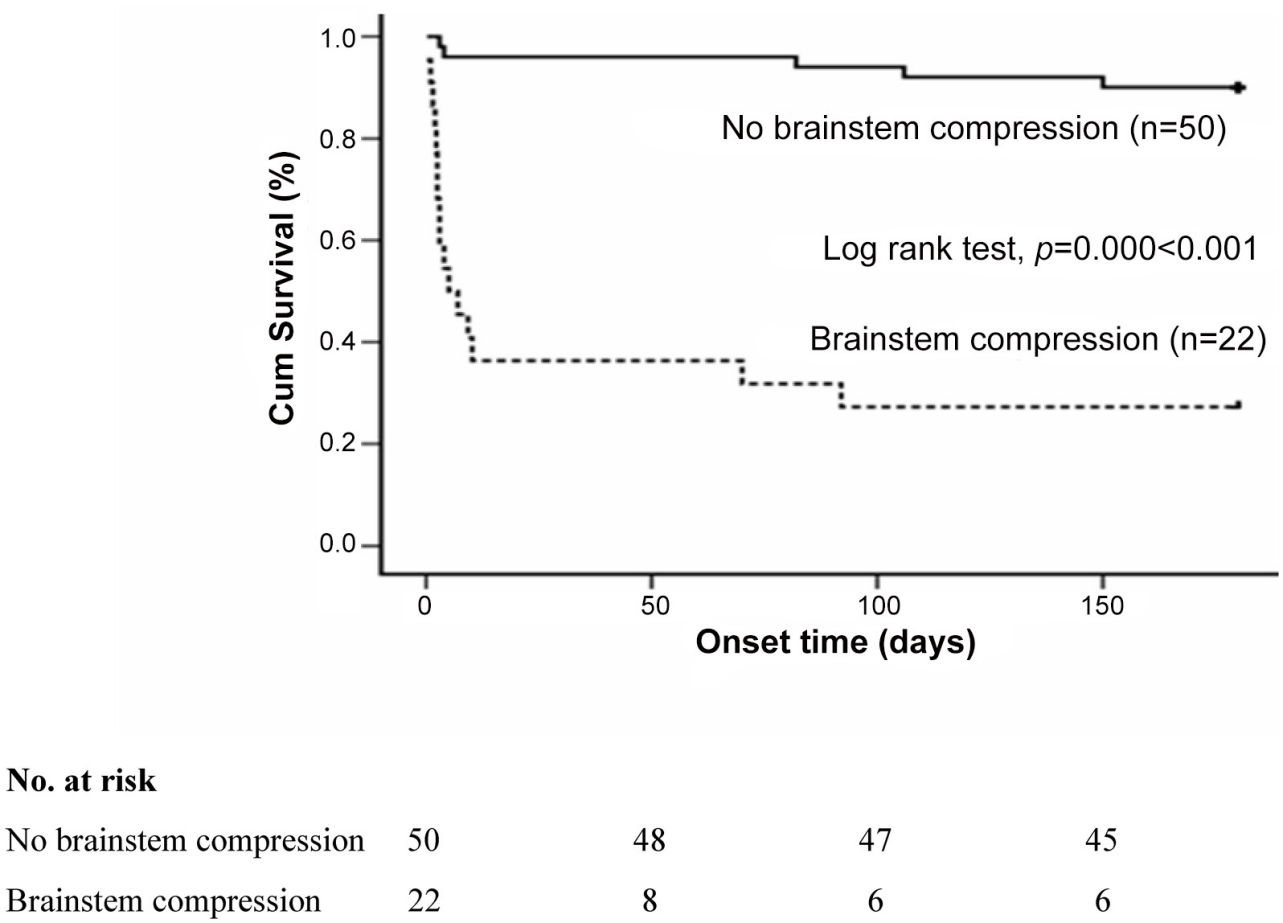
Kaplan-Meier survival analysis for rate of six-month mortality.

Median onset time of six-month mortality was 5 days in the radiological brainstem compression group (p < 0.001) and the HRs for the onset time of six-month mortality was 13.1 compared with patients without brainstem compression (95% CI, 4.7 to 36.3, p < 0.001) (Table 3). The Kaplan-Meier curves show significant rate of six-month mortality in brainstem compression group (Log-rank test, p < 0.001) (Fig 1).

**Table 3:** The brainstem and no brainstem compression group of Kaplan-Meier and Cox regression analysis.

	Kaplan-Meier survival analysis			Cox regression analysis		
Group	No. of patients	Median onset time (days)	*p* value	HRs	95% CI	*p* value
No brainstem compression	50	-		13.1	4.7–36.3	0.000***
Brainstem compression	22	5	0.000***			

Note: HRs = Hazard Ratios; CI = Confidence Interval;

***p<0.001

## Discussion

Identifying effective predictors of intermediate-term mortality in patients with CH is important. This study showed that radiological brainstem compression (HRs = 23.5) was a strong independent predictor of mortality within six-month in patients with spontaneous CH. Patients with radiological brainstem compression were about 23 times more likely to die within 6 months after CH than those without radiological brainstem compression.

Brainstem compression by cerebellar hematoma is a specific and severe complication of CH and may contribute to neurological deterioration, impaired consciousness, and death [15]. Indeed, radiological brainstem compression was predictive of early neurological deterioration and poor outcome [12]. We also showed that radiological brainstem compression were strong predictors of first-week mortality [4].

Moreover, radiological brainstem compression was associated with unfavorable long-term outcomes and mortality [6, 7]. However, in some previous studies, predictors of long-term mortality were not the same as those of early- and intermediate-term mortality. For example, Pong et al. [8] showed that GCS 8 was predictive of 30-day mortality and Louis et al. [5] reported that GCS scores < 8, acute hydrocephalus, and IVH independently predicted death within 3 months after CH onset.

Only four studies have investigated predictive factors of intermediate- and long-term mortality or outcomes after CH onset [5–8] (Table 4). Louis et al. [5] reported admission systolic blood pressure > 200 mm Hg, hematoma size >3cm, visible brain stem distortion, and acute hydrocephalus predict intermittent-term (3-month) poor outcome while mortality within 3-month could be predicted by GCS scores < 8, acute hydrocephalus, and IVH independently after CH onset. Dolderer and colleagues [6] followed 75 patients with CH and the long-term mortality rate after a mean follow-up time of 49 ± 34 months was 48%. They demonstrated that the factors associated with unfavorable long-term outcome were decreased level of consciousness, clinical signs of brainstem compression, hydrocephalus, radiological brainstem compression, intubation, and surgical intervention. Moreover, the factors associated with mortality were clinical signs of brainstem compression, hydrocephalus, ventilation, surgical decompression, GCS score, and hematoma size. In another study, 57 surgically treated patients with CH were followed for at least 12 months, and a total of 14 patients died (25%). Initial neurological condition, level of consciousness, radiological brainstem compression, and a tight posterior fossa were predictive of poor clinical outcome [7]. In a recent study by Pong et al. [8] that followed 72 Chinese patients, age 75 years was an independent predictor of poor functional status at 6 months (34 of 60 (56.7%) patients survived). However, no independent predictor of intermediate- or long-term mortality was identified in these previous studies except report by Louis et al. [5], and the current study is the first report to reveal radiological brainstem compression being independent predictor of mortality within six-month after CH onset.

**Table 4:** Overview of risk or predictive factors of intermediate- and long-term mortality or poor outcome in patients with cerebellar hemorrhage.

Study (first author, year)	Patient number	Mean age (year)	Mean follow-up time (month)	Predictors or associated factors of mortality/poor outcome
Louis (2000)^[^ ^5^ ^]^	94	70 (27–89)	> 3	**Predictors for intermittent poor outcome** (Admission systolic blood pressure > 200 mm Hg, hematoma size >3cm, visible brain stem distortion, and acute hydrocephalus). **Predictors for mortality within 3-month** (Abnormal corneal and oculocephalic response, GCS<8, motor response less than localization to pain, acute hydrocephalus and IVH).
Dolderer (2004)^[^ ^6^ ^]^	75	66.9 ± 11.4	49.0 ± 34.0	**Factor s related with long-term poor outcome** (Consciousness level, hydrocephalus, clinical signs of brainstem compression, intubation and surgical intervention). **Factor s related with long-term mortality** (Clinical signs of brainstem compression, hydrocephalus, ventilation, surgical management, GCS score, hematoma size).
Dammann (2011)^[^ ^7^ ^]^	57	64.4 (30–82)	34.0 (12–107)	**Predictors for long-term poor outcome** (Initial neurological condition, level of consciousness, radiological brainstem compression, and a tight posterior fossa).
Pong (2012)^[^ ^8^ ^]^	72	67.5 ± 12.3	57	**Predictor for poor functional status at 6 months** (Age ≥75).
Present study	72	65.2 ± 16.1	>6	**Factors related with mortality within six-month** (Coagulopathy, GCS ≤8, initial BS levels ≥140 mg/dl, maximum hematoma diameter ≥3 cm, radiological brainstem compression).**Predictor for mortality within six-month**(Radiological brainstem compression).

Note: GCS = Glasgow Coma Scale, IVH = intraventicular hemorrhage, BS = blood glucose/sugar.

A few published studies investigating patients with intracranial hemorrhage (ICH) mention predictors of intermediate- and long-term mortality can be used for reference. However no or very few cases of CH were included in these studies because of the relatively low prevalence. These studies were summarized in Table 5 [11, 16–23]. For example, Daverat and co-workers [16] reported that age correlated with six-month survival rates. The level of consciousness and age were the best related to mortality at 6 and 12 months [17]. Inagawa et al.[18] reported that increasing age and GCS, rebleeding, and hematoma volume were significantly associated with the 2-year survival rate. Hemphill et al.[23] reported that age, hemorrhagic volume, level of consciousness, IVH, and infratentorial location were independent predictors of 1-year mortality. Nilsson et al [19] showed that the initial level of consciousness, age, and hematoma location were independent predictors of 1-year mortality after ICH. Vermeer et al.[11] reported that age 65 years was the only predictor of vascular death.

**Table 5:** Overview of intermediate- and long-term mortality in patients with intracranial hemorrhage.

Study (first author, year)	Patient number	Mean age (year)	Mean follow-up time (month)	Predictors of intermediate- and long-term mortality
Daverat (1991)^[^ ^16^ ^]^	166	61	6	**Predictor for six-month mortality** (Age)
Hardemark (1999)^[^ ^17^ ^]^	203	60–65	6 and 12	**Predictors for six- and twelve-month mortality** (GCS and age)
Inagawa (2000)^[^ ^18^ ^]^	267	Unknown	24	**Predictors for 2-year mortality** (GCS score, age, rebleeding, and hematoma volume)
Nilsson (2002)^[^ ^19^ ^]^	341	74	12	**Predictors for 1-year mortality** (Initial level of consciousness, age and hematoma location)
Vermeer(2002)^[^ ^11^ ^]^	243	64 ±13	66	**Predictor for long-term mortality** (age > 65)
Garibi (2002)^[^ ^20^ ^]^	185	65	6	**Predictor for six-month mortality** (GCS, preictal status in relation to activity daily activity and age)
Saloheimo(2006)^[^ ^21^ ^]^	140	65.9	84	**Predictor for long-term mortality** (Smoking, age and diabetes)
Zia (2009)^[^ ^22^ ^]^	474	72.7±11.8	36	**Predictor of 3-year mortality** (Male sex, high age, central and brainstem hemorrhage location, intraventricular hemorrhage, increased volume, and decreased consciousness level)
Hemphill (2009)^[^ ^23^ ^]^	243	65.0 ± 15.0	6 and 12	**Predictor of 1-year mortality** (GCS, hematoma volume, presence of IVH, infratentorial origin and age ≥80)

Note: GCS = Glasgow Coma Scale.

Reviewing the above mentioned studies, the increasing age was the most common predictor of long-term mortality of ICH. However, age 65 years was not predictive of six-month mortality in the current study. In addition to a higher prevalence of radiological brainstem compression in CH, the relatively younger mean age (65.2 ± 16.1 years) of the patients in this study might contribute to the difference in findings. In general, the condition of the patient and complications associated with increasing age may have larger effects on long-term mortality, especially in older patients.

The results of this study must be viewed in light of its limitations. First, the case number is relatively small because of the low incidence of CH compared with other types of ICH despite an 8-year period collection. Second, it might be slightly subjective to define radiological brainstem compression on the basis of imaging findings alone and without brainstem-evoked potentials or electroencephalography; however, it is difficult to perform them in such an emergent condition. Third, no information was available regarding medications prescribed during the follow-up period, such as anticoagulant agents. The inability to exclude the effects of these differences may have affected outcome results. However, hypertension is the major cause of CH and is reported to account for 60–89% of all cases. The likelihood of anticoagulant medications being prescribed to patients with hemorrhagic stroke was very low compared to patients with ischemic stroke. Therefore, the confounding resulting from side effects of anticoagulant agents, such as recurrent hemorrhage, was unlikely in this study. Last, the bias caused by the single-center retrospective hospital-based analysis posed limitations in generalization. Although a prospective design would be better, it would take many years to collect an adequate number. Further prospective analysis is need in the future.

## Conclusions

Radiological brainstem compression was a strong predictive factor for mortality within six-month in patients with CH and patients with radiological brainstem compression were about 23 times more likely to die within 6 months after CH than those without radiological brainstem compression. These findings may facilitate the identification of predictive factors for intermediate-term mortality after CH, thereby helping clinicians and patients.
